# Psychometric evaluation of two short versions of the Revised Child Anxiety and Depression Scale

**DOI:** 10.1186/s12888-020-2444-5

**Published:** 2020-02-05

**Authors:** Leonie Klaufus, Eva Verlinden, Marcel van der Wal, Mia Kösters, Pim Cuijpers, Mai Chinapaw

**Affiliations:** 1 Public Health Service Amsterdam, Department of Epidemiology, Health Promotion, and Health Care Innovation, Nieuwe Achtergracht 100, Amsterdam, Netherlands; 2 Amsterdam UMC, Vrije Universiteit Amsterdam, Department of Public and Occupational Health, Amsterdam Public Health research institute, De Boelelaan 1117, Amsterdam, Netherlands; 3 Vrije Universiteit Amsterdam, Department of Clinical, Neuro, and Developmental Psychology, Amsterdam Public Health research institute, Van der Boechorststraat 7, Amsterdam, Netherlands

**Keywords:** Anxiety, Depression, Psychometrics, Child, Adolescent, Screening, Questionnaires

## Abstract

**Background:**

Anxiety and depression are common in children and adolescents, which can be detected via self-report questionnaires in non-clinical settings like the school environment. Two short versions of the Revised Child Anxiety and Depression Scale (i.e., RCADS-25 and RCADS-20) seem to be feasible for administering at schools. The present study evaluated the psychometric properties of the RCADS-25 and RCADS-20 used as screening instruments for anxiety and depression in a general population of schoolchildren and adolescents.

**Methods:**

The RCADS-25 was completed by 69,487 schoolchildren and adolescents aged 8 to 18. The RCADS-25 and RCADS-20 broad anxiety scales are equal (15 items), but there are two versions of the major depressive disorder (MDD) scale: the RCADS-25 MDD scale (10 items) and the RCADS-20 MDD scale (5 items). The three scales were assessed on structural validity, internal consistency, test-retest reliability, criterion validity, and hypotheses for construct validity.

**Results:**

The RCADS-25/RCADS-20 broad anxiety scale demonstrated a sufficient structural validity (CFI = 0.98, TLI = 0.99, RMSEA = 0.03, SRMR = 0.03), internal consistency (alpha = 0.82), test-retest reliability (ICC = 0.73), criterion validity (AUC = 0.79), and all four hypotheses concerning construct validity were confirmed. The RCADS-25 MDD scale demonstrated a sufficient test-retest reliability (ICC = 0.70) and three out of four hypotheses concerning construct validity were confirmed, but its structural validity was suspect (CFI = 0.89, TLI = 0.94, RMSEA = 0.09, SRMR = 0.06). The RCADS-20 MDD scale demonstrated a sufficient structural validity (CFI = 0.97, TLI = 0.97, RMSEA = 0.08, SRMR = 0.04) and internal consistency (alpha = 0.72). Two out of four hypotheses concerning construct validity were confirmed. The test-retest reliability (ICC = 0.60) was insufficient. Since both MDD scales showed shortcomings, the shortening of the RCADS-25 MDD scale was re-examined post hoc by principal component and reliability analyses. The result was an MDD scale with seven items.

**Conclusions:**

The RCADS-25/RCADS-20 broad anxiety scale is valid and reliable for screening schoolchildren and adolescents, but the RCADS-25 and RCADS-20 MDD scales showed shortcomings. An MDD scale of seven items showed acceptable psychometric properties.

## Background

Anxiety and depression are common conditions in children and adolescents and can have a devastating impact on their lives. Prevalence rates vary from 11 to 25% for an anxiety disorder and from 3 to 8% for a depressive disorder [1, 2]. When left untreated, anxiety and depression might recur or lead to other problems later in life, like substance abuse or dependence, suicidal behavior, educational underachievement, unemployment, and early parenthood [3–6]. To prevent these problems, it is important that children and adolescents with anxiety and depression are detected and offered an intervention as early as possible [7, 8].

Early detection of anxious and depressed children and adolescents can be achieved by proactive screening in a non-clinical setting such as the school environment via self-report screening questionnaires [9, 10]. To be feasible in a school environment, these questionnaires should be affordable and as short as possible [9], especially when these need to be included in a screening battery. And importantly, they should demonstrate good psychometric properties for screening in a general population.

The Revised Child Anxiety and Depression Scale (RCADS) [11] seems to have an advantage in feasibility over other self-report questionnaires regarding anxiety and depression in children and adolescents. The RCADS is freely available, in contrast to questionnaires like the Screen for Child Anxiety Related Emotional Disorders-Dutch version (SCARED-NL) [12] or the Children’s Depression Inventory-second version (CDI-2) [13]. In addition, the RCADS measures both anxiety and depression, and the original RCADS of 47 items [11] has been shortened to versions with 25 items (i.e., RCADS-25) [14] and 20 items (i.e., RCADS-20) [15]. By way of comparison, the SCARED-NL measures anxiety with 69 items, and the CDI-2 measures a depressive syndrome with 28 items, which are 97 items in total.

Previous studies have demonstrated promising psychometric properties of the RCADS-25. The RCADS-25 was developed by shortening five separate anxiety scales of the RCADS-47 (i.e., separation anxiety disorder [SAD], social phobia [SOC], generalized anxiety disorder [GAD], obsessive-compulsive disorder [OCD], and panic disorder [PD]) to one broad anxiety scale by bi-factor modeling: three items per anxiety scale were selected that loaded strongly on a general broad anxiety factor, strongly and simply on the specific anxiety factors, and were not synonymous [14]. The result was a broad anxiety scale consisting of 15 items. The original ten items measuring a major depressive disorder (MDD) were left unchanged [14]. The broad anxiety scale showed a sufficient reliability (alpha = 0.86; omega hierarchical = 0.71) in a school-based sample (*N* = 1060; age = 6–18) [14]. The MDD scale showed a sufficient reliability (alpha = 0.76–0.79 [11, 14]; ICC = 0.82 [16]) and convergent validity compared to the CDI (r = 0.7) [11] in school-based samples (*N* = 246–1641; age = 6–18).

The RCADS-20 has also demonstrated promising psychometric properties in previous research. In the RCADS-20, the broad anxiety scale of the RCADS-25 was left unchanged, but the number of MDD items were reduced by half (five items remaining) in conformance with the MDD scale of the Spanish RCADS-30 [15, 17]. MDD scale reduction was based on ranked item loadings, corrected item-total correlations, and content validity [17]. Previous research has indicated the existence of a general broad anxiety factor and a separate MDD factor with five items (CFI = 0.96, TLI = 0.93, RMSEA = 0.05) in a general sample (*N* = 501; age = 8–14) [15]. The MDD scale with five items showed a sufficient reliability (alpha = 0.72) and convergent validity compared to the CDI (r = 0.6) in a school-based sample (*N* = 544; age = 10–17) [17].

Due to the advantage of feasibility of the RCADS short versions in a school setting, the RCADS-25 has been translated into Dutch. In order to be able to use it as a screening instrument for anxiety and depression in schoolchildren and adolescents, it is important to examine whether the promising psychometric properties of the RCADS-25 and the RCADS-20 can be replicated and to add research on the remaining psychometric properties. The present study offers a comprehensive overview of the structural validity, internal consistency, test-retest reliability, criterion validity, and hypotheses testing for construct validity of the RCADS-25 and the RCADS-20 subscales according to the internationally consensus-based COSMIN taxonomy, definitions, and quality criteria of measurement properties [18–20]. The COSMIN taxonomy, definitions, and quality criteria were used to prevent confusion about the meaning of the measurement properties and to follow the preferred statistical methods [18]. The psychometric properties of the RCADS-25 and RCADS-20 subscales were examined in, to the best of our knowledge, the largest general sample of schoolchildren and adolescents to date.

## Methods

### Participants and procedures

Participants were 70,777 schoolchildren and adolescents aged 8 to 18 who completed the RCADS-25 online at school; 69,487 students completed the RCADS-25 without too many missing values (i.e., three or fewer missing items on the broad anxiety scale and two or fewer missing items on the MDD scale [21]). The completion of the RCADS-25 was part of a screening program for health and socio-emotional problems, which was executed at 345 schools from September 2017 to August 2018. These schools voluntarily registered for the screening program and were located in urban and rural areas throughout the Netherlands.

Additionally, a convenience sample of the above-mentioned schools was approached for extra data collection procedures by youth health care professionals who carried out the screening program at the schools. The selection of schools was based on their screening program planning and on including a variation of urban and rural schools in the present study. If schools granted permission, informed consents and assents for the extra data collection procedures were sent to parents and children or adolescents.

The additional data collection procedures varied per school, class, age, and level of education, aiming to obtain maximum variation in the various subsamples for examining different psychometric properties. To examine test-retest reliability, 277 participants (response rate = 77%) completed the RCADS-25 a second time under the same test conditions as 2 to 4 weeks before when completing the RCADS-25 for the first time during the usual screening. To examine criterion validity, 110 other participants (response rate = 17%) had a semi-structured interview after the usual screening (i.e., the Schedule for Affective Disorders and Schizophrenia for School-Age Children Present and Lifetime Version [K-SADS-PL] [22, 23]). Within this group of interviewees, maximum variation was aimed at in scores below, between, and above the 90th and 95th percentiles of the RCADS-25 subscales in order to avoid spectrum bias; however, this procedure might have influenced the response rate (which is further discussed in limitations and strengths section). To examine hypotheses for construct validity, another 545 participants completed an extra questionnaire during the usual screening: 269 participants (response rate = 84%) completed the SCARED-NL [12, 24, 25] and 276 participants (response rate = 89%) completed the CDI-2 [13, 26, 27]. Table 1 shows the demographic characteristics of the participants in the various study samples. All participants received a small gift (€1–5 in value); participating schools received a gift voucher of €25–50 depending on the number of participating classes.

**Table 1:** Demographic characteristics of the various study samples

	Total sample	Subsample	Subsample	Subsample	Subsample
	(*N* = 69,487)	(*N* = 277)	(*N* = 110)	(*N* = 269)	(*N* = 276)
	Structural validity, internal consistency	Test-retest reliability	Criterion validity	Hypotheses testing SCARED-NL	Hypotheses testing CDI-2
Gender					
Girls (%)	49.7	48.0	55.5	52.0	46.0
Age					
M (SD)	14.2 (1.3)	12.5 (2.7)	13.0 (2.1)	12.7 (2.6)	11.9 (2.3)
Education^a^					
Elementary (%)	2.0	56.0	23.6	43.1	56.9
Lower Vocational (%)	41.8	17.0	27.3	8.2	26.8
Intermediate Vocational (%)	0.5	17.7	8.2	4.5	2.5
Higher General Secondary (%)	20.2	0.4	15.5	20.1	7.2
Pre-university (%)	18.7	9.0	25.5	24.2	6.5

*SCARED-NL* Screen for Child Anxiety Related Emotional Disorders-Dutch version, *CDI-2* Children’s Depression Inventory-second version, *M* Mean, *SD* Standard deviation

^a^ Percentages may not add up to 100% due to the omission of less common or mixed education levels, or missing values

### Measures

#### Revised Child Anxiety and Depression Scale - short versions (RCADS-25, RCADS-20)

The RCADS-25 [14] is a short self-report questionnaire for children and adolescents aged 8 to 18 that measures broad anxiety through 15 items and MDD through ten items in accordance with the DSM-IV descriptions of anxiety disorders and MDD. All 15 anxiety items and five out of ten MDD items were used to examine the psychometric properties of the RCADS-20 [15]. Items are scored on a four-point Likert scale: 0 (never), 1 (sometimes), 2 (often), and 3 (always), resulting in a range of total scores from 0 to 45 for the broad anxiety scale, from 0 to 30 for the RCADS-25 MDD scale with ten items (MDD-10), and from 0 to 15 for the RCADS-20 MDD scale with five items (MDD-5); higher total scores indicate a more severe level of anxiety or MDD.

#### Schedule for Affective Disorders and Schizophrenia for School-Age Children Present and Lifetime version (K-SADS-PL)

The K-SADS-PL [22, 23] is a semi-structured interview in which symptoms of DSM-IV diagnoses are assessed in children and adolescents aged 6 to 18. The K-SADS-PL starts with an unstructured introductory interview, followed by a screen interview, and ends with five supplementary interviews that may or may not be conducted depending on the scores in the screening interview. Symptoms are scored by presence and severity on a 0 to 3 rating scale: 0 indicates no information is available, 1 indicates a symptom is not present, 2 indicates a subthreshold presence of a symptom, and 3 indicates a threshold presence of a symptom. Previous studies have found a sufficient inter-rater agreement of the screening interview (99.7%) and of assigning present diagnoses (98% [22]; kappa = 0.76 for MDD and kappa = 0.84 for any anxiety disorder [28]); a good test-retest and reliability (kappa = 0.90 for MDD; kappa = 0.80 for any anxiety disorder [22]), and a moderate to sufficient convergent validity (r = 0.45–0.47, *p* < 0.01 for MDD [29]; B = 4.18, p < 0.01 in linear regression analyses concerning any anxiety diagnosis [30]).

In the present study, three trained interviewers conducted the screening interview and, regardless of the scores on the screening interview, the supplementary interviews of the current episodes of the panic disorder, separation anxiety disorder, avoidant disorder/social phobia, overanxious/generalized anxiety, obsessive-compulsive disorder, and depressive disorders. The three interviewers were blinded to the RCADS-25 scores. They had a Bachelor’s or Master’s degree in Psychology or Psychiatric nursing, and they regularly held peer meetings to give feedback and to promote uniformity in the administration of the interviews. All interviews were audio-recorded; five randomly selected recordings per interviewer were scored a second time by an external fourth trained interviewer to determine the inter-rater agreement. The observed inter-rater agreement of the screening interview ranged from 87 to 100% for the anxiety disorders and was 87% for MDD. The observed inter-rater agreement of derived diagnoses ranged from 87 to 100% for the anxiety disorders and was 100% for the MDD diagnosis.

#### Screen for Child Anxiety Related Emotional Disorders-Dutch version (SCARED-NL)

The SCARED-NL [12] is a self-report questionnaire for children and adolescents aged 7 to 19 that measures childhood anxiety disorders in conformance with the DSM-IV-TR (i.e., separation anxiety, panic disorder, specific phobia, social phobia, obsessive-compulsive disorder, posttraumatic and acute stress disorder, and generalized anxiety disorder) through 69 items. Respondents are asked to indicate how often they experience the described situation: “never or almost never”, “sometimes”, or “often”, which are scored as 0, 1, or 2 respectively. By adding up these item scores, a total score is calculated that ranges from 0 to 138; higher total scores indicate a more severe level of anxiety. Concerning these total scores, previous studies have demonstrated a sufficient test-retest reliability (ICC = 0.81) [31] and a moderate to good convergent validity (r = 0.67–0.88) [31–34]. In the present study, alpha was 0.96 for the total score.

#### Children’s Depression Inventory-2 (CDI-2)

The Dutch translation of the revised CDI [26, 27] was used, which is a self-report screening instrument for a depressive syndrome in children and adolescents aged 8 to 21. It consists of 28 items, with each item presenting three sentences that describe different severity levels of a symptom; respondents are asked to report which sentence describes their situation best. The severity levels per item are scored as 0, 1, and 2, which can be added up to a total score that ranges from 0 to 56; a higher total score indicates a more severe level of a depressive syndrome. Previous research findings regarding the psychometric properties of the total score have revealed moderate to sufficient results in a general population concerning the test-retest reliability (r = 0.60) and convergent validity (r = 0.77) [35]. In the present study, alpha was 0.85 for the total score.

### Analyses

#### Missing data and selection bias

Missing data for RCADS-25 scores were handled in accordance with the RCADS-25 Child Version Scoring Program 3.1 [21]. This program prescribes mean replacement when there are three or fewer missing items on the broad anxiety scale and two or one missing items on the MDD-10 scale. Concerning the MDD-5 scale, mean replacement was performed when there was one missing item. Missing data for the SCARED-NL and the CDI-2 were handled the same way: mean replacement was performed when there was no more than one missing value per five items per subscale. Cases with more than the allowable missing items were excluded from analyses.

Potential selection bias in the additional data collection procedures was examined through multilevel logistic regression analyses. These analyses were adjusted for school and class by means of a random intercepts model. Odds ratios were calculated for children and adolescents who did and did not complete the additional data collection procedures on the one hand, and gender, age group (i.e., 8–12 years and 13–18 years), and scores below and above the 90th percentile of the RCADS-25 scales on the other, since these scale scores were skewed to the right. This is in line with current practice, as children and adolescents scoring above the 90th percentile are invited for further investigation by school nurses and physicians. The 90th percentiles of the RCADS-25 broad anxiety and MDD-10 scale were determined in a national representative sample with respect to gender, age, region, ethnicity, household size, and social class, based on Statistics Netherlands data from 2017 [36] (see Additional file 1).

#### Psychometric properties

Anxiety and MDD are considered to be different constructs in the DSM. Therefore, structural validity, internal consistency, test-retest reliability, criterion validity, and hypotheses about construct validity were assessed for the RCADS-25/RCADS-20 broad anxiety scale, RCADS-25 MDD-10 scale, and RCADS-20 MDD-5 scale separately. These psychometric properties were analyzed in the preferred order of importance according to COSMIN [37].

##### Structural validity

Confirmatory factor analyses (CFAs) were conducted to examine the unidimensionality of the separate subscales. Unidimensionality refers to the extent that item responses on a scale are driven by the latent trait the scale purports to measure [38]. First, a one-factor model fit was examined with the broad anxiety data, the MDD-10 data, and the MDD-5 data. Second, a bi-factor model fit was examined with the broad anxiety data only, since the broad anxiety scale was developed by exploratory bi-factor modeling [14]. In the bi-factor model, all anxiety items were allowed to load on a general broad anxiety factor as well as on one of five orthogonal group factors (i.e., SAD, SOC, GAD, OCD, and PD) in accordance with the description of Ebesutani and colleagues [14]. In both the one-factor and bi-factor model fit tests, item responses were indicated as ordered, the diagonally weighted least squares model estimation was used, and mean and variance adjusted test statistics were calculated. Model fit was assessed through four indices: a scaled comparative fit index (CFI), a scaled Tucker-Lewis index (TLI), a scaled root mean square error of approximation (RMSEA), and a standardized root mean square residual (SRMR). A CFI or TLI close to or higher than 0.95, and an RMSEA close to or lower than 0.06 or an SRMR close to or lower than 0.08 were considered as indicators of a good fit [39]. Further, an RMSEA between 0.06 and 0.1 was considered as mediocre [40], and CFI or TLI values between 0.90 and 0.95 were considered as an acceptable fit [41]; all remaining scores were considered as indicators of an unacceptable fit.

In case of a lack of a one-factor model fit and a sufficient bi-factor model fit, it was examined whether the broad anxiety scale was unidimensional in essence. Essential unidimensionality was examined by calculating the omega hierarchical, which is a statistic that estimates the proportion of variance in raw scores attributable to the general factor [42]; an omega hierarchical of at least 0.8 was considered as an indicator of a scale that is unidimensional in essence. Essential unidimensionality can also be assessed by the explained common variance (ECV), which refers to “the ratio of variance explained by the general factor divided by the variance explained by the general factors and the group factors” [43]. However, the interpretation of ECV, if used as a unidimensional measure in the context of a bi-factor model, is moderated by the percentage of uncontaminated correlations (PUC) [43]. PUC is a statistic of the percentage of inter-item correlations accounted for by the general factor only [44]. The PUC of the broad anxiety scale is considered high (i.e., 0.86) [43], since PUC values greater than 0.8 indicate a low risk of bias when treating a multidimensional scale as unidimensional [43]. Since the PUC is high, the ECV was considered as less important as an indicator of unidimensionality [43].

##### Internal consistency

Internal consistency was assessed by calculating a Cronbach’s alpha per subscale. An alpha equal to or greater than 0.70 was considered as sufficient [20]. However, in case of a bi-factor model fit to the anxiety data, Cronbach’s alpha can be misleading [42]. In that case, omega hierarchical in combination with omega total can be regarded as appropriate model-based reliability indicators [43]. Omega total refers to the proportion of the total variance attributable to the general and group factors. Omega hierarchical and omega total were considered sufficient if they were equal to or greater than 0.8 [44].

##### Test-retest reliability

Test-retest reliability was assessed by calculating an intraclass correlation coefficient (ICC) and its 95% confident interval. ICCs were calculated by the use of a single rater, absolute agreement, and a two-way mixed effect model. ICCs of 0.70 or higher were considered as sufficient [20].

##### Criterion validity

Criterion validity was assessed by calculating receiver operating curves (ROCs). One ROC was calculated for the broad anxiety scale in comparison with any anxiety disorder according to the K-SADS-PL (i.e., panic disorder, separation anxiety disorder, avoidant disorder/social phobia, overanxious/generalized anxiety, and/or obsessive-compulsive disorder). Two ROCs were calculated for the MDD-10 and MDD-5 scales in comparison with depressive disorders according to the K-SADS-PL.

##### Hypotheses testing for construct validity

Construct validity was assessed by testing four hypotheses based on inferences from previous studies. It was determined a priori that construct validity of the separate subscales would be sufficient if at least three out of four hypotheses were confirmed [20].

Our first hypothesis was that girls have higher mean scores than boys on the broad anxiety scale, MDD-10 scale, and MDD-5 scale [1–3, 6]. This hypothesis was tested by multilevel linear regression analyses, adjusted for school and class by the use of a random intercepts and random slope model. Mean differences were expected of at least one point on the broad anxiety scale [14], one point on the MDD-10 scale [11, 14], and 0.5 point on the MDD-5 scale [15].

Our second hypothesis was a positive correlation of 0.6 to 0.7 between the broad anxiety scale and the two MDD scales, since anxiety and depression have been found to be comorbid [2, 45]. These correlations are comparable to results of previous RCADS research [15, 16].

Our third hypothesis was a positive correlation of at least 0.7 between the broad anxiety scale and the SCARED-NL, of at least 0.65 between the MDD-10 scale and the CDI-2, and of at least 0.6 between the MDD-5 and the CDI-2. These correlations were expected, since related constructs are measured, and previous studies have reported comparable results [11, 17]. Moreover, fewer items decrease reliability, and the test-retest reliability of the CDI-2 has shown to be moderate [35].

Our fourth hypothesis was a positive correlation of at least 0.6 between the broad anxiety scale and the CDI-2, and between the MDD scales and the SCARED-NL; in addition, these correlations were expected to be lower than the correlations between the broad anxiety scale and the SCARED-NL and between the MDD scales and CDI-2. This hypothesis was based on the fact that slightly different constructs are measured that were expected to correlate highly.

#### Software

All analyses were conducted in SPSS version 21, with the exception of the CFAs, which were performed using the lavaan package in RStudio version 1.1.463, and of the multilevel regression analyses, which were performed in Stata Intercooled 15.

## Results

### Descriptive statistics and selection bias

Table 2 shows the descriptive statistics of the RCADS-25 and RCADS-20 broad anxiety and MDD scales. Children and adolescents who participated in the additional data collection procedures scored above the 90th percentile of the RCADS-25 broad anxiety or MDD-10 scale more often than those who did not participate (OR = 4.86, 95% CI = 3.15–7.50). Participation rates did not differ between boys and girls (OR = 1.11, 95% CI = 0.82–1.50), nor between age groups (OR = 0.38, 95% CI = 0.13–1.12).

**Table 2:** Descriptive statistics of RCADS-25 and RCADS-20 subscales by age group and gender

			RCADS-25		RCADS-20^a^
			Broad anxiety	MDD-10	MDD-5
Age group	Gender	*N*	M (SD)	M (SD)	M (SD)
8–12	Boys	2429	5.9 (4.9)	5.0 (3.4)	1.8 (1.8)
	Girls	2643	7.4 (5.6)	5.4 (3.8)	2.4 (2.0)
	Total	5072	6.7 (5.4)	5.2 (3.6)	2.0 (1.9)
13–18	Boys	32,530	4.3 (3.9)	4.4 (3.3)	1.5 (1.7)
	Girls	31,881	6.6 (5.1)	5.8 (4.0)	2.3 (2.1)
	Total	64,415	5.5 (4.7)	5.1 (3.7)	1.9 (2.0)
8–18	Boys	34,959	4.4 (4.0)	4.4 (3.3)	1.5 (1.7)
	Girls	34,524	6.7 (5.1)	5.8 (4.0)	2.3 (2.1)
	Total	69,487	5.6 (4.7)	5.1 (3.7)	1.9 (2.0)

*RCADS* Revised Child Anxiety and Depression Scale, *MDD* Major depressive disorder, *M* Mean, *SD* Standard deviation

^a^ The broad anxiety scale of the RCADS-20 is equal to the broad anxiety scale of the RCADS-25

### Structural validity

Table 3 shows the CFA results of the one-factor model fit with the broad anxiety data, the MDD-10 data, and the MDD-5 data, and of the bi-factor model fit with the broad anxiety data. The broad anxiety data best fitted a bi-factor model rather than a one-factor model. In addition, the MDD-10 data did not seem to fit a one-factor model, but the MDD-5 data did fit the one-factor model.

**Table 3:** Model fit results from CFAs of RCADS-25 and the RCADS-20 subscales (*N* = 69,487)

	RCADS-25			RCADS-20^a^
	Broad anxiety		MDD-10	MDD-5
	One-factor	Bi-factor	One-factor	One-factor
CFI^b^	0.81	0.98	0.89	0.97
TLI^b^	0.92	0.99	0.94	0.97
RMSEA^b^	0.08	0.03	0.09	0.08
SRMR	0.07	0.03	0.06	0.04

*CFAs* Confirmatory factor analyses, *RCADS* Revised Child Anxiety and Depression Scale, *MDD* Major depressive disorder, *CFI* Comparative fit index, *TLI* Tucker-Lewis index, *RMSEA* Root mean square error of approximation, *SRMR* Standardized root mean square residual

^a^ The broad anxiety scale of the RCADS-20 is equal to the broad anxiety scale of the RCADS-25

^b^ The scaled indices are used to correct for non-normality of the data

Table 4 shows the factor loadings of the items per subscale in the one-factor and bi-factor models. In the bi-factor model, some broad anxiety items showed higher standardized loadings on a group factor than on the common broad anxiety factor (i.e., “afraid of own at home”, “tremble or shake”, “what other people think of me”, and “do things over and over again”). However, an omega hierarchical of 0.84 and the PUC of 0.86 suggested the existence of a strong general factor.

**Table 4:** One-factor and bi-factor solutions of RCADS-25 and RCADS-20 subscales (N = 69,487)

	RCADS-25							RCADS-20
	One-factor	Bi-factor						One-factor
Abbreviated item content		BA	SAD	GAD	PD	SOC	OCD	
Broad anxiety								
Afraid of own at home	0.50	0.47	0.83					
Afraid of crowded places	0.59	0.62	0.07					
Scared if I have to sleep on my own	0.59	0.56	0.43					
Something will happen to family	0.58	0.57		0.31				
I think about death	0.54	0.55		0.16				
Something bad will happen	0.72	0.72		0.55				
Suddenly become dizzy or faint	0.50	0.50			0.27			
Tremble or shake	0.54	0.53			0.93			
Suddenly get a scared feeling	0.72	0.76			0.03			
Worry when done poorly	0.67	0.64				0.25		
What other people think of me	0.71	0.58				0.66		
Worry I might look foolish	0.73	0.61				0.54		
Do things over and over again	0.43	0.40					0.44	
Have to think special thoughts	0.63	0.61					0.43	
Do things in just the right way	0.60	0.58					0.53	
MDD								
Feels sad or empty	0.75							0.81
Feels nothing is much fun anymore	0.68							0.71
Has trouble sleeping	0.60							
Has problems with appetite	0.45							
Has no energy for things	0.65							0.55
Cannot think clearly	0.66							0.62
Feels worthless	0.76							0.83
Feels like does not want to move	0.52							
Feels tired a lot	0.69							
Feels restless	0.64							

*RCADS* Revised Child Anxiety and Depression Scale, *BA* Common broad anxiety factor, *SAD* Separation anxiety disorder group factor, *GAD* Generalized anxiety disorder group factor, *PD* Panic disorder group factor, *SOC* Social phobia group factor, *OCD* Obsessive-compulsive disorder group factor, *MDD* Major depressive disorder

### Internal consistency and test-retest reliability

Table 5 shows the results of the reliability analyses. The internal consistency of the broad anxiety scale and the MDD-5 scale were sufficient according to Cronbach’s alpha. However, the broad anxiety data fitted a bi-factor model, and therefore the omega hierarchical of 0.84 and omega total of 0.93 were considered better reflections of the true reliability of the broad anxiety scale than Cronbach’s alpha. Cronbach’s alpha was not calculated for the RCADS-25 MDD-10 scale, since the data did not fit a one-factor model, and unidimensionality is a prerequisite for its interpretation [46]. In addition, the test-retest reliability was sufficient for the broad anxiety scale and MDD-10 scale, but too low for the MDD-5 scale (ICC = 0.60).

**Table 5:** Reliability and hypotheses testing for construct validity of the RCADS-25 and RCADS-20 subscales

			RCADS-25		RCADS-20^a^
			Broad anxiety	MDD-10	MDD-5
Reliability					
Internal consistency	(*N* = 69,487)	Cronbach’s alpha	0.82	n.a.^b^	0.72
Test-retest reliability^c^	(*N* = 277)	ICC (95% CI)	0.73 (0.66–0.78)	0.70 (0.62–0.76)	0.60 (0.52–0.67)
Hypotheses testing					
Hypotheses about correlation SCARED-NL	(N = 269)	r (95% CI)	0.84 (0.80–0.87)	0.68 (0.61–0.74)	0.66 (0.58–0.72)
Hypotheses about correlation CDI-2	(N = 276)	r (95% CI)	0.66 (0.59–0.72)	0.66 (0.58–0.72)	0.59 (0.51–0.67)

*RCADS* Revised Child Anxiety and Depression Scale, *MDD* Major depressive disorder, *n.a* Not applicable, *ICC* Intraclass correlation coefficient, *SCARED-NL* Screen for Child Anxiety Related Emotional Disorders-Dutch version, *CDI-2* Children’s Depression Inventory-second version

^a^ The broad anxiety scale of the RCADS-20 is equal to the broad anxiety scale of the RCADS-25

^b^ Cronbach’s alpha was not calculated for the RCADS-25 MDD-10 scale, since the data did not fit a one-factor model, and unidimensionality is a prerequisite for its interpretation

### Criterion validity

**Fig. 1:**
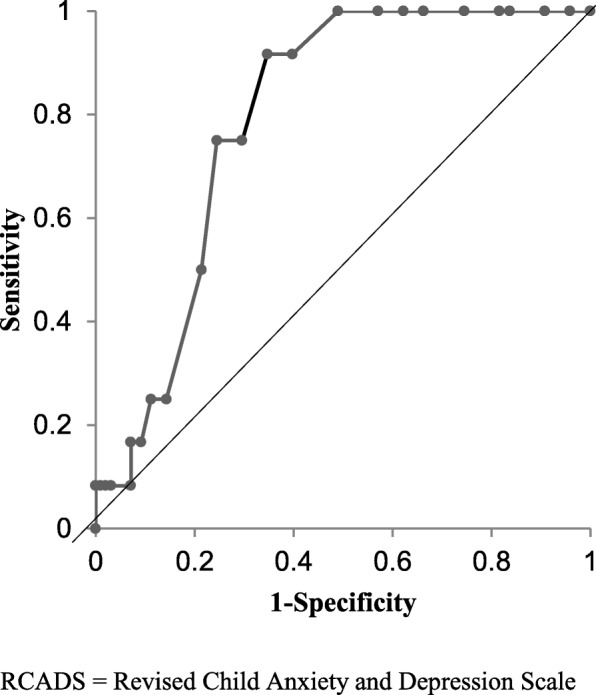
Receiver operating characteristic curve for the RCADS-25/RCADS-20 broad anxiety scale RCADS = Revised Child Anxiety and Depression Scale

Of 110 children and adolescents in the criterion validity subsample, 62% had a broad anxiety score below the 90th percentile, 21% had a score between the 90th and 95th percentiles, and 17% had a score above the 95th percentile. With regard to the MDD-10 scale, these percentages were 66, 19, and 16% respectively. According to the K-SADS-PL, 11 participants (10%) had symptoms of one or two anxiety disorders, one (1%) had symptoms of MDD, and one (1%) had symptoms of both an anxiety disorder and MDD. Figure 1 shows the ROC of the broad anxiety scale with an area under the curve (AUC) of 0.79. A cutoff score of ≥12 corresponded to a sensitivity of 0.92 and a specificity of 0.65; a cutoff score of ≥14 corresponded to a sensitivity of 0.75 and a specificity of 0.76. ROCs for the MDD scales were not calculated, since only two participants met the criteria for MDD.

### Hypotheses testing for construct validity

All four hypotheses concerning the broad anxiety scale, three out of four hypotheses concerning the MDD-10 scale, and two out of four hypotheses concerning the MDD-5 scale were confirmed. Girls had higher scores than boys: mean differences were 2.2 (95% CI = 2.1–2.3) on broad anxiety, 1.3 (95% CI = 1.3–1.4) on the MDD-10, and 0.7 (95% CI = 0.7–0.8) on the MDD-5 scale. A positive correlation was found of 0.64 (95% CI = 0.64–0.65) between the broad anxiety and MDD-10 scale, and of 0.64 (95% CI = 0.63–0.64) between the broad anxiety and MDD-5 scale. Table 5 shows the correlation between the RCADS-25 and RCADS-20 subscales on the one hand and the SCARED-NL and CDI-2 on the other. The correlations between the broad anxiety scale and the SCARED-NL, and between the MDD-10 and the CDI-2 were as expected, but the correlation between the MDD-5 and the CDI-2 was just below 0.6 (r = 0.59). In addition, the correlations between the broad anxiety scale and the CDI-2, and between the MDD scales and the SCARED-NL were as expected, but surprisingly, the correlations between the MDD scales and the SCARED-NL were higher than the ones between the MDD scales and the CDI-2.

### Post hoc analyses

Since the findings suggested shortcomings of the MDD-10 and MDD-5 scales, post hoc, the possibility to shorten the MDD-10 scale was re-examined so that it meets all criteria. Therefore, the total sample was randomly divided into a training set and a validation set (*N* = 34,644 and *N* = 34,843 respectively).

In the training set, principal component and reliability analyses were conducted on the MDD-10 data. One after the other the following items were deleted: “has problems with appetite”, “feels like does not want to move”, and “has no energy for things”, based on their lowest ranked item loadings, on their lowest corrected item-total correlation, and on their content validity conform the analyses of Sandín and colleagues [17]. Concerning content validity, items that were considered to be core depression symptoms were retained a priori, while items that were more similar in content were deleted. For example, the items “feels tired a lot” and “has no energy for things” concerned the same symptom of MDD in the DSM-IV; “has no energy for things” was deleted since its ranked item loading and corrected item-total correlation were lower. The result was an MDD scale with seven items (MDD-7).

In the validation set, it was examined whether this MDD-7 scale met the following criteria: 1. a sufficient one-factor model fit (CFI and TLI ≥ 0.9, RMSEA < 1, SRMR ≤0.8); 2. a sufficient internal consistency (Cronbach’s alpha ≥0.7). In addition, in the various subsamples it was examined whether the MDD-7 scale met the following criteria: 3. a sufficient test-retest reliability (ICC ≥ 0.7); 4. a correlation of at least 0.6 between the MDD scale and the CDI-2; 5. a correlation of at least 0.6 between the MDD scale and the SCARED-NL, but lower than between the MDD scale and the CDI-2.The MDD-7 data showed a mediocre one-factor model fit (CFI scaled = 0.93, TLI scaled = 0.95, RMSEA scaled = 0.10, SRMR = 0.06), a sufficient Cronbach’s alpha of 0.78, a sufficient ICC of 0.70, and a correlation with the CDI-2 of r = 0.68; however, the correlation with the SCARED-NL remained the same (r = 0.68). Deleting two items compared to three items downgraded the results of the one-factor model fit and the test-retest reliability to just below sufficient levels. Deleting four items downgraded the test-retest reliability to insufficient levels.

## Discussion

The aim of the current study was to examine the psychometric properties of the RCADS-25 and RCADS-20 scales when applied as screening instruments in a non-clinical population of schoolchildren and adolescents.

### RCADS-25/RCADS-20 broad anxiety scale

The structural validity, internal consistency, test-retest reliability, and criterion validity of the RCADS-25/RCADS-20 broad anxiety scale all proved sufficient, and the four hypotheses to examine construct validity were confirmed.

A bi-factor model fit with the broad anxiety data, as described by Ebesutani and colleagues [14], was confirmed. Despite this multidimensional model fit, the scale seems unidimensional in essence. Unidimensionality was shown by the high PUC (> 0.8) and the high omega hierarchical (> 0.8), with the latter being even higher than originally reported [14]. The ratio between the omega hierarchical and the omega total (i.e., 0.84 / 0.93 = 0.90) shows that 90% of the total anxiety score represents the variance of the broad anxiety factor. Therefore, the use of the anxiety total score seems substantiated [42].

In addition to unidimensionality indicators, omega hierarchical and omega total can be regarded as reliability coefficients. Both coefficients were greater than 0.8, and the omega total was even greater than 0.9, which indicates that the broad anxiety scale is highly reliable. The test-retest reliability was sufficient, which indicates that the error variance is small compared to the variance between individuals. Therefore, the broad anxiety scale is applicable to screening purposes.

The criterion validity was adequate, with an AUC comparable with previous research [14]. Since all hypotheses were confirmed, the construct validity of the broad anxiety scale proved sufficient as well.

### RCADS-25 MDD-10 and RCADS-20 MDD-5

The psychometric properties of the RCADS-25 MDD-10 and RCADS-20 MDD-5 scales showed mixed results.

Concerning the RCADS-25 MDD-10 scale, the test-retest reliability was sufficient and three out of four hypotheses about construct validity were confirmed, but its structural validity was insufficient. One-factor model fit was rejected, because the CFI was too low, and the TLI and RMSEA showed a mediocre fit. Lack of one-factor model fit suggests that other latent constructs are measured in addition to MDD. The test-retest reliability of the MDD-10 scale was sufficient, but lower than previous research has shown [16]. Also, three out of four hypotheses were confirmed. The one hypothesis that was rejected concerned an expected lower correlation between the MDD-10 and the SCARED-NL than between the MDD-10 and the CDI-2: this correlation was higher. Therefore, it is possible that the MDD-10 scales measures anxiety as well as MDD, although this suggestion is in contrast to earlier findings [15].

The structural validity and internal consistency of the RCADS-20 MDD-5 scale were sufficient, but its test-retest reliability was insufficient and only two out of four hypotheses about construct validity were confirmed (although one hypothesis was close to acceptable). The fewer number of items of the MDD-5 scale improved its structural validity, as model fit with a one-factor model was sufficient. Although the internal consistency remained acceptable, the reduced number of items degraded the test-retest reliability to insufficient. Also, the construct validity was uncertain, since the correlation between the MDD-5 scale and the CDI-2 was just below 0.6 (r = 0.59); still, this correlation seems fairly strong, and 0.6 is just an arbitrary predefined cutoff value. However, this correlation was lower than the correlation between the MDD-5 and the SCARED-NL (r = 0.66).

There are some possible explanations for these shortcomings. An equal or higher correlation between the MDD scales and the SCARED-NL than between the MDD scales and the CDI-2 might indicate an inadequate discriminant validity of the MDD scales or of the CDI-2. The latter possibility is mentioned in previous studies [35], but not in all [47, 48]. Another possible explanation is that anxiety and depression are not completely distinct constructs during child development [49]. This explanation might be consistent with the finding that both anxiety and depression are part of a general distress component with additional specific anxiety and depression symptoms [50]. Also, there are indications that anxiety precedes the development of depression [6, 51]. The mixed psychometric findings might also be the result of differential item functioning: children with the same level of MDD might score differently on the corresponding English and Dutch items, due to an inaccurate translation or to differences in interpretation.

To overcome the shortcomings of both MDD scales, it was re-examined post hoc whether the MDD-10 could be shortened to an MDD scale with sufficient psychometric properties. To shorten the MDD-10 scale, the analyses of Sandín and colleagues [17] were repeated. The result was an MDD scale of seven items (MDD-7) with just sufficient psychometric properties. The three items that were removed from the MDD-10 scale were: “has problems with appetite”, “feels like does not want to move”, and “has no energy for things”. These findings are partly consistent with former research. In the research of Sandín and colleagues [17], the items “has problems with appetite” and “feels like does not want to move” were removed as well. In the study of Chorpita and colleagues [11], these items showed rather low factor loadings (i.e., 0.28 and 0.26 respectively). A possible explanation for the low impact of the item “has problems with appetite” might be the late appearance of the symptom in the development of MDD during childhood [51]. The removal of the item “has no energy for things” was in contrast to the study of Sandín and colleagues [17]: this item was retained in the MDD-5 scale and showed a factor loading of 0.47. However, in the study of Skoczeń and colleagues [15], this factor loading was less than 0.30. Retaining the item “has no energy for things” leaded to an insufficient one-factor model fit and test-retest reliability, while the MDD-7 scale resulted in a mediocre structural validity, a sufficient internal consistency and test-retest reliability, and a confirmation of three out of four hypotheses. The one hypothesis that was rejected concerned an expected lower correlation between the MDD-7 and the SCARED-NL than between the MDD-7 and the CDI-2: these correlations were equal.

### Limitations and strengths

Limitations of the present study include the limited sample size to examine criterion validity (*N* = 110). Due to the low prevalence of MDD a sample of at least 400 children and adolescents is needed for a high sensitivity, a power of at least 80%, and a *p*-value of less than 0.05 [52]. Such a sample size was practically unfeasible when conducting time-consuming diagnostic interviews. Furthermore, all subsamples were convenience samples. This limitation was compensated for by including a maximum variation in schools, classes, ages, levels of education, and — in the criterion validity subsample — in the levels of anxiety and MDD, leading to the lower response rate in the latter subsample. Despite this maximum variation, selection bias might have occurred, which might lead to spectrum bias in screening research if the included groups mainly show much higher or lower levels of anxiety and depression compared tot the general population. Participants in the subsamples more often showed a score above the 90th percentile on the RCADS-25 subscales; however, the prevalences of anxiety and MDD diagnoses in the criterion validity subgroup were comparable with prevalences reported in previous research [2], and the response rates were high in the other subsamples. These high response rates were considered a strength of the present study, in addition to the very large sample size for examining structural validity and internal consistency, and the completeness in psychometric properties examinations.

### Recommendations for screening practice

The present study has several implications for screening practice. All total subscale scores were skewed to the right while the RCADS items are formulated on a threshold level, which might hamper distinguishing children and adolescents on the level of anxiety and MDD at the lower end of the scales. However, for screening purposes, only making a distinction at the higher end of the scale is of interest. Therefore, professionals are recommended to use the separate scales to select children and adolescents scoring at the high end, e.g., above the 90th percentile; on the anxiety scale, this percentile of the total group equals the cutoff score ≥ 12 with a high sensitivity and a reasonable specificity. Since both MDD-10 and 5 scales showed some inadequate psychometric properties, we advice using the MDD-7 in practice.

### Recommendations for future research

Future research should replicate the psychometric properties of the MDD-7 scale as a screening instrument for MDD in a general population. In addition, future research could examine whether the explained variance by one factor increases by the reformulation of the deleted MDD items, or by developing and adding some new MDD items. Also, future studies could investigate a higher order model fit or a bi-factor model fit with the combined anxiety and MDD data, since all MDD scales showed equal or lower correlations with the CDI-2 than with the SCARED-NL. Finally, future research could examine differential item functioning across age, since the probability of endorsements of the separation anxiety items might be higher at a younger age, and the probability of the endorsement of the MDD item “has problems with appetite” might be higher at an higher age.

## Conclusions

The RCADS-25/RCADS-20 anxiety scale is reliable and valid for screening schoolchildren and adolescents, but the MDD scales of both the RCADS-25 and RCADS-20 show some shortcomings. Therefore, an MDD scale with seven items is recommended, although replication studies and additional research are needed to examine its psychometric properties.

## Supplementary information

**Additional file 1. **Percentiles of RCADS-25 and RCADS-20 subscales in a representative general Dutch sample (*N* = 1319).

## Data Availability

The dataset is available from the corresponding author on reasonable request.

## Abbreviations

AUC: Area under the curve
CDI-2: Children’s Depression Inventory-second version
CFA: Confirmatory factor analysis
CFI: Comparative fit index
COSMIN: Consensus-based Standards for the selection of health Measurement INstruments
DSM: Diagnostic and Statistical Manual of Mental Disorders
ECV: Explained common variance
GAD: Generalized anxiety disorder
ICC: Intraclass correlation coefficient
K-SADS-PL: Schedule for Affective Disorders and Schizophrenia for School-Age Children Present and Lifetime version
MDD: Major depressive disorder
OCD: Obsessive-compulsive disorder
PD: Panic disorder
PUC: Percentage of uncontaminated correlations
RCADS: Revised Child Anxiety and Depression Scale
RMSEA: Root mean square error of approximation
ROC: Receiver operating curve
SAD: Separation anxiety disorder
SCARED-NL: Screen for Child Anxiety Related Emotional Disorders-Dutch version
SOC: Social phobia
SRMR: Standardized root mean square residual
TLI: Tucker-Lewis index
