# Supplementary material for: Psychometric evaluation of two short versions of the Revised Child Anxiety and Depression Scale
Source: BMC Psychiatry. 2020 Feb 5;20:47. doi: 10.1186/s12888-020-2444-5 (PMC7003441; doi:10.1186/s12888-020-2444-5)
Supplement: Supplementary file 1 — Additional file 1. Percentiles of RCADS-25 and RCADS-20 subscales in a representative general Dutch sample (N = 1319). [file 12888_2020_2444_MOESM1_ESM.docx]

Additional file 1

**Percentiles of RCADS-25 and RCADS-20 subscales in a representative^a^ general Dutch sample (N = 1,319)**

|  |  |  | RCADS-25 | |  |  | RCADS-20^b^ | |
| --- | --- | --- | --- | --- | --- | --- | --- | --- |
|  |  |  | Broad anxiety | | MDD-10 | | MDD-5 | |
| Age | Gender | N | 90 | 95 | 90 | 95 | 90 | 95 |
| 8-12 | Boys | 334 | 11.58 | 13.47 | 9.46 | 10.79 | 4.36 | 5.17 |
|  | Girls | 335 | 12.27 | 15.33 | 8.97 | 10.92 | 4.15 | 4.88 |
|  | Total | 669 | 11.79 | 14.33 | 9.26 | 10.83 | 4.26 | 4.97 |
| 13-18 | Boys | 318 | 9.36 | 12.00 | 8.68 | 9.87 | 4.07 | 4.95 |
|  | Girls | 332 | 14.17 | 16.44 | 12.56 | 15.19 | 5.81 | 6.95 |
|  | Total | 650 | 11.89 | 15.50 | 10.11 | 13.91 | 4.93 | 6.30 |
| All | Boys | 652 | 11.03 | 13.29 | 9.17 | 10.37 | 4.23 | 5.04 |
|  | Girls | 667 | 13.00 | 16.13 | 10.45 | 13.36 | 4.88 | 6.18 |
|  | Total | 1,319 | 11.82 | 15.08 | 9.72 | 11.79 | 4.59 | 5.70 |

RCADS = Revised Child Anxiety and Depression Scale; MDD = major depressive disorder.

^a^ Representative with respect to gender, age, region, ethnicity, household size, and social class.

^b^ The broad anxiety scale of the RCADS-20 is equal to the broad anxiety scale of the RCADS-25.
